# The Cost of an Operating Room Minute for Heart Valve Procedures

**DOI:** 10.36469/9898

**Published:** 2015-03-09

**Authors:** Eugene A. Grossi, Matthew Moore, Peter J. Mallow, John A. Rizzo

**Affiliations:** 1 Cardiothoracic Surgery NYU Langone Medical Center, New York, NY USA; 2 Global Health Economic Strategy, Edwards Lifesciences Inc., Irvine, CA USA; 3 CTI Clinical Trial and Consulting Services, Cincinnati, OH USA; 4 Economics and Preventative Stony Brook University, Stony Brook, NY USA

**Keywords:** premier hospital database, retrospective analysis, heart valve disease, cost analysis, operating room

## Abstract

**Background:** Heart valve disease is very common, with approximately 5 million people diagnosed with this disease annually in the United States. There has been substantial innovation in the technologies and techniques of surgical repair and replacement over the past decade. However, there is little information that allows the potential time savings associated with these technologies and techniques to be quantified from the hospital perspective.

**Objectives:** The study objective was to estimate the variable cost per operating room (OR) minute in valvular procedures – aortic valve replacement (AVR), mitral valve replacement (MVR) and mitral valve repair (MVRepair) – and determine if there is a difference in OR cost per minute between traditional sternal versus less invasive right thoracotomy surgical techniques.

**Methods:** The Premier database was queried from 2007 to 2011 for patients undergoing AVR, MVR, or MVRepair. Patients were identified using the International Classification of Diseases, 9th Revision (ICD-9) procedure codes. Propensity score matching created cohorts adjusted for patient differences and surgical approach –any sternal incision (Sternal) or right thoracotomy (RT). Regression analysis was performed to estimate the OR cost per minute based on heart valve procedure.

**Results:** There were 2,656 heart valve procedures – 1,604 AVR, 434 MVR and 618 MVRepair – that met the inclusion criteria. The mean OR cost per minute for AVR procedures was $26.49 and $25.16 (p <0.01) for Sternal and RT, respectively. MVR procedures by surgical approach had a mean OR cost per minute of $25.66 and $25.00 and (p NS) for Sternal and RT, respectively. MVRepair procedures by surgical approach had a mean OR cost per minute of $25.17 and $24.40 and (p NS) for Sternal and RT, respectively. The overall estimate of the OR cost per minute for valvular procedures was $25.99.

**Conclusions:** Quantifying the variable cost of an OR minute from a multi-institution database provides researchers with an important benchmark to use in economic evaluations of valvular procedures.

### INTRODUCTION

Heart valve disease is common, with approximately 5 million people diagnosed with this disease annually.[1] Currently, there are no drug therapies that can cure heart valve disease, and surgery is the definitive treatment for symptomatic patients and recommended for some asymptomatic patients.[2,3] These surgeries are among the 20 most commonly-performed procedures in the United States, with 120,000 performed in 2011.[4] Collectively, heart valve surgeries ranked 8th in aggregate hospital costs ($6.07 billion) and 1st in average cost per hospital stay ($53,400) in 2011.[4] The most commonly-performed heart valve surgeries include the following three procedures: aortic valve replacement (AVR), mitral valve replacement (MVR) and mitral valve repair (MVRepair).

The technology of heart valve surgery is evolving rapidly.[5-7] Innovations in heart valve surgeries have focused on improved delivery mechanisms such as sutureless valves and rapid deployment systems. These innovations make the surgery easier to perform and may improve operating room (OR) efficiency. Although these new innovations are often more expensive, the cost of the technology must be balanced against efficiency gains that may reduce costs in the OR. Quantifying the cost of OR time associated with these surgeries would help inform budgetary decisions in today's cost-conscious hospital environment regarding the deployment of new innovations.

However, there is surprisingly little evidence quantifying the cost of OR time for specific procedures, even though overall OR costs comprise one-third of the hospital budget.[8] It is expected that the cost of OR time would be linked to the procedure, with coronary artery bypass graft (CABG) costing more than an inguinal hernia repair, for example.[9] Literature searches on OR costs reveal numerous articles comparing the cost of hospitalizations following traditional surgical approaches versus more recent techniques,[10-12] or studies that describe ways to improve OR efficiency by streamlining processes linked to room set-up or surgical supply management.[13,14]

What is lacking, however, is reliable evidence quantifying the cost of using an OR per minute, including the cost of set-up, performing the procedure, and post-procedure room turnover. One clinical expert has argued that there are no reliable published data on OR costs per minute.[9] Macario based this argument in part on the study conducted by Shippert.[15] That study reported a mean cost of $62 per OR minute, with a wide range of $21.80 to $133.10 per minute. The study employed a qualitative survey design and focused on cosmetic procedures. Nevertheless, it illustrated that reducing OR time can have an important economic impact.[15]

From a research and evaluation perspective, quantifying hospital costs down to the minute level will enable hospitals and health service researchers to determine the economic impact of the OR when performing cost-effectiveness analyses or budget impact analyses. With these metrics, hospitals will be better able to understand the entire cost implications of introducing new technological innovations into the OR.

The purpose of this study is to estimate the cost per OR minute of three isolated non-robotic valvular procedures: AVR, MVR and MVRepair. Using a national hospital database to perform our analysis, we also sought to determine if cost per OR minute for these procedures varied by any sternal incision (Sternal) or right thoracotomy (RT) approaches and to provide researchers with information for economic evaluations using real-world data.

## METHODS

### Data Source

The Premier hospital database – a large-sample hospital database widely used and cited regularly in health services and clinical outcomes research – was employed as the data source for this study.[16] This database contains complete patient billing, hospital cost, and coding histories from more than 600 healthcare facilities throughout the United States. The data used in this study were extracted from more than 25 million inpatient discharges and 175 million hospital outpatient visits from acute care facilities, ambulatory surgery centers, and clinics across the nation.

A protocol describing the analysis objectives, criteria for patient selection, data elements of interest, and statistical methods was submitted to the New England Institutional Review Board (NEIRB) and exemption was obtained (NEIRB # 13-203).

### Patient Selection

All patients were 18 years or older at the time of the visit and admitted for valve replacement or repair as the primary procedure. International Classification of Diseases, 9$^{th}$ Revision Clinical Modification (ICD-9-CM) codes were used to identify the primary procedure. The following codes were used: AVR 35.21, 35.22; MVR 35.23, 35.24; and MVRepair 35.12. Patients with CABG or whose surgery utilized robotic technology were excluded.

A set of expert rules were developed to help analyze the free text fields of the charge master billing files of the Premier database in order to categorize surgical approach as either Sternal or RT. Appendix A displays these expert rules for procedure identification and attrition.

For all eligible patients, elements describing adverse events, hospital cost, surgery time, length of stay and readmissions were obtained from the database. Cost analysis reflected the actual cost of the procedure to the hospital. The pre-operative All Patient Refined Diagnosis Related Groups (APR-DRG) severity level was used as an index of clinical comorbidity. The 3M APR DRG Classification System is a widely adopted proprietary risk adjustment classification tool, which uses information from routine claims data to produce valid and reliable severity measurement and risk adjustment scores.[17] It is used to account for differences related to an individual's severity-of-illness or risk-of-mortality in large datasets. Comorbid conditions that might influence procedure selection or outcomes of interest, such as previous organ transplantation, the presence of pulmonary disease or diabetes mellitus, were obtained using ICD-9 diagnosis and procedure codes. Information on socio-demographic characteristics and health insurance status was also included, as were descriptors of the care setting, namely census region, urban or rural setting, teaching hospital status, and facility bed count.

### OR Cost and Time Definition

OR cost per minute was calculated for each patient by dividing the sum of the reported OR costs by the sum of OR reported time. Costs were assigned to the OR department category by the individual hospitals and reviewed by Premier for appropriateness and accuracy. The OR cost units captured by Premier hospitals were those items specific to the patient visit (i.e. variable costs) and included: hospital-supplied staff time, anesthesia services (including recovery services), blood transfusion services and OR recovery services. OR time was reported by the individual hospitals in the chargemaster of the Premier hospital database. Premier hospitals report the OR time and associated costs attributable to the specific procedure. It does not capture specific procedure time (i.e. incision to close time). Thus, it is appropriate to view the OR time as the total time the hospital used for the procedure including any pre-procedure setup and post-procedure room recovery time attributable to the patient. Each individual hospital was responsible for documenting the OR time. These time increments were summed at the patient visit level to estimate the total OR time per visit. All visits were assessed for outliers based on OR time within each procedure group. The top and bottom 5% of patient visits were removed. This step eliminated visits with no OR time reported or excessively long periods of time.

#### Analysis

The goal of the propensity matching analysis was twofold: 1) to find pairs of patients receiving Sternal and RT procedures within the specific valvular procedure who share like propensities for candidacy for the procedure, based on the matching variables; and 2) to maximize the number of matched patients, while ensuring that cohorts were not significantly different with respect to relevant characteristics.

A SAS® macro from the Mayo Clinic was utilized which employed "nearest-neighbor matching" on the estimated propensity scores to choose Sternotomy matches for the patients who had the RT approach.[18] Propensity scores were calculated for receipt of RT procedures for each of the patients included in the analysis based on a multivariable logistic regression model. Patients receiving the RT technique were then matched to patients having a Sternal procedure with a 1:1 ratio exactly (caliper=0) on their gender. Study subjects were simultaneously matched within (±) 5 years on age and within a value of 0.0001 on their propensity for having a Sternal procedure. Patient characteristics and comorbid conditions used in the propensity score model included: race, marital status, region and insurance, severity index, angina, dysrhythmias, chronic obstructive pulmonary disease (COPD), depression, extensive aortic atherosclerosis, kidney disease, previous coronary angioplasty, myocardial infarction (acute or old) and other coronary artery disease.

Once the matched pairs were obtained, to assess the extent to which the propensity matching reduced confounders, the distribution of several variables before and after matching were compared, including age, gender, race, insurance type, health status, region, and comorbid conditions among the patients in the cohorts. Group comparisons were made using chi-square tests.

A generalized linear model (GLM) with a gamma distribution and a log link function was used to further adjust for differences in hospital characteristics (teaching versus non-teaching, urban versus rural and bed count) for the propensity matched sample. The GLM calculated the corresponding least square means of overall cost for the hospital stay within each matched cohort. All costs were adjusted to 2012 dollars using the Medical Care Component of the Consumer Price Index.

### RESULTS

A total of 40,719 patients underwent an AVR, MVR or MVRepair procedure between 2007 and 2011, and the procedural technique (Sternal or RT) was determined by the expert rule. The final attrition step identified 2,656 valvular procedures of which 1,604; 434; and 618 were AVR, MVR and MVRepair, respectively (Figure 1). Table 1 reports the patient characteristics of the propensity-matched cohorts by valvular surgery type. There were no statistically significant differences between Sternal and RT procedures for any of the characteristics examined.

**Figure 1.** Attrition Diagram

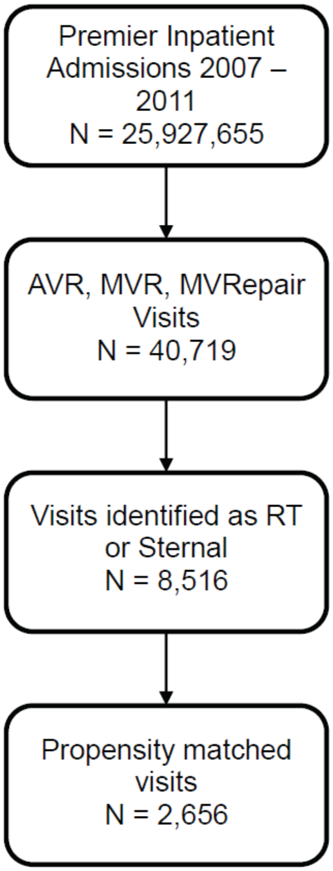

Note: Of the 2,656 propensity matched visits, 1,604 were AVR, 434 were MVR, and 618 were MVRepair.

Table 2 reports the OR cost per minute by type of heart valve procedure and surgical approach after matching. The overall AVR mean cost per OR minute was $27.8 (SD $9.40). AVR procedures by surgical approach had a mean OR cost per minute of $29.3 and $26.5 (p-value<0.001) for Sternal and RT, respectively. The overall MVR mean cost per OR minute was $30.7 (SD $12.50). MVR procedures by surgical approach had a mean OR cost per minute of $31.4 and $30.2 (p-value NS) for Sternal and RT, respectively. The overall MVRepair mean cost per OR minute was $28.7 (SD $10.30). MVRepair procedures by surgical approach had a mean OR cost per minute of $30.3 and $27.4 (p-value<0.001) for Sternal and RT, respectively.

Table 3 reports the hospital characteristics after matching by valvular procedure and surgical approach. For AVR, MVR, and MVRepair there were statistically significant differences between Sternal and RT surgical approach for region, teaching status, and bed count. Location was statistically significant for MVRepair, but not AVR and MVR. The least squares mean estimates for OR cost per minute by valvular procedure and surgical approach are provided in Table 4. Separate regression analyses for each valvular procedure were performed using the respective propensity matched cohorts and controlling for hospital characteristics. AVR procedures by surgical approach had a mean OR cost per minute of $26.49 and $25.16 (p-value<0.01) for Sternal and RT, respectively. MVR procedures by surgical approach had a mean OR cost per minute of $25.66 and $25.00 (p-value NS) for Sternal and RT, respectively. MVRepair procedures by surgical approach had a mean OR cost per minute of $25.17 and $24.40 (p-value NS) for Sternal and RT, respectively. A fourth regression model performed with the entire sample population estimated the overall mean OR cost per minute of $25.99 while controlling for valvular procedure, surgical approach, and hospital characteristics.

**Table 1.** Patient Characteristics After Matching

| | AVR (n = 1,604) | | | MVR (n = 434) | | | MVRepair (n = 618) | | |
|---|---|---|---|---|---|---|---|---|---|
| | Sternal (n=802) | RT (n=802) | | Sternal (n=217) | RT (n=217) | | Sternal (n=309) | RT (n=309) | |
| Category | (%) | (%) | p-value | (%) | (%) | p-value | (%) | (%) | p-value |
| **Age** | | | | | | | | | |
| 18 - 29 | 0.3 | 0.7 | 0.942 | 0.4 | 0.4 | 0.900 | 2.0 | 2.5 | 0.733 |
| 30 - 39 | 2.6 | 2.4 | | 1.2 | 0.8 | | 2.7 | 2.2 | |
| 40 - 49 | 5.1 | 4.8 | | 3.8 | 4.6 | | 11.7 | 12.7 | |
| 50 - 59 | 12.3 | 13.3 | | 15.3 | 12.6 | | 22.3 | 19.3 | |
| 60 - 69 | 22.6 | 21.6 | | 29.1 | 30.3 | | 25.7 | 27.9 | |
| 70 - 79 | 35.8 | 36.2 | | 39.1 | 37.2 | | 30.1 | 28.9 | |
| 80 or older | 21.3 | 21.2 | | 11.1 | 14.2 | | 5.6 | 6.6 | |
| **Race** | | | | | | | | | |
| Caucasian | 86.3 | 85.2 | 0.877 | 68.6 | 71.3 | 0.253 | 71.4 | 77.3 | 0.172 |
| African American | 2.6 | 2.7 | | 6.5 | 9.6 | | 9.8 | 6.6 | |
| Hispanic | 0.5 | 0.4 | | 4.2 | 4.2 | | 2.2 | 1.2 | |
| Other | 10.5 | 11.6 | | 20.7 | 14.9 | | 16.6 | 14.9 | |
| **Gender** | | | | | | | | | |
| Female | 39.9 | 39.9 | 1.000 | 57.9 | 57.9 | 1.000 | 45.0 | 45.0 | 1.000 |
| Male | 60.2 | 60.2 | | 42.2 | 42.2 | | 55.0 | 55.0 | |
| **Marital Status** | | | | | | | | | |
| Married | 63.2 | 62.8 | 0.742 | 60.9 | 62.5 | 0.936 | 63.8 | 63.8 | 1.000 |
| Unmarried | 34.7 | 34.6 | | 38.3 | 36.8 | | 34.0 | 34.0 | |
| Other/Unknown | 2.1 | 2.6 | | 0.8 | 0.8 | | 2.2 | 2.2 | |
| **Insurance** | | | | | | | | | |
| Commercial | 1.1 | 1.4 | 0.946 | 0.4 | 0.4 | 1.000 | 3.2 | 3.2 | 1.000 |
| Medicare | 66.9 | 66.8 | | 69.4 | 69.4 | | 49.9 | 49.9 | |
| Medicaid | 1.0 | 1.2 | | 3.1 | 3.1 | | 2.9 | 2.9 | |
| Managed Care | 29.4 | 29.2 | | 25.7 | 25.7 | | 39.9 | 39.9 | |
| Other | 1.6 | 1.4 | | 1.5 | 1.5 | | 4.2 | 4.2 | |
| **Health Status** | | | | | | | | | |
| APR-DRG Severity Level (1, 2) | 41.9 | 41.5 | 0.850 | 19.5 | 18.0 | 0.654 | 39.6 | 36.2 | 0.313 |
| APR-DRG Severity Level (3, 4) | 58.1 | 58.5 | | 80.5 | 82.0 | | 60.4 | 63.8 | |

AVR: aortic valve replacement; MVR: mitral valve replacement; MVRepair: mitral valve repair; RT: right thoracotomy; APR-DRG: All Patient Refined Diagnosis Related Groups

**Table 2.** OR Costs per Minute After Matching by Valvular Procedure

| | AVR | | | | MVR | | | | MVRepair | | | |
|---|---|---|---|---|---|---|---|---|---|---|---|---|
| | All | Sternal | RT | p-value | All | Sternal | RT | p-value | All | Sternal | RT | p-value |
| **Mean** | $27.80 | $29.30 | $26.50 | <0.001 | $30.70 | $31.40 | $30.20 | NS | $28.70 | $30.30 | $27.40 | <0.001 |
| **SD** | $9.40 | $9.20 | $9.40 | | $12.50 | $11.50 | $13.40 | | $10.30 | $11.20 | $9.30 | |
| **Median** | $27.60 | $28.70 | $25.20 | | $28.90 | $29.20 | $28.70 | | $28.30 | $28.70 | $27.80 | |
| **25th Percentile** | $19.70 | $22.00 | $17.90 | | $20.70 | $23.30 | $19.80 | | $20.40 | $21.70 | $19.80 | |
| **75th Percentile** | $33.40 | $34.60 | $32.60 | | $37.20 | $37.60 | $35.60 | | $35.20 | $38.20 | $33.50 | |

P-value shown compares Sternal vs. RT for the specific valvular procedure. OR: operating room; AVR: aortic valve replacement; MVR: mitral valve replacement; MVRepair: mitral valve repair; RT: right thoracotomy; SD: standard deviation

**Table 3.** Hospital Characteristics After Matching by Valvular Procedure

| | AVR | | | | |
|---|---|---|---|---|---|
| | Sternal | | RT | | |
| | Visits (n = 802) | Hospitals (n = 65) | Visits (n = 802) | Hospitals (n = 52) | |
| Category | (%) | (%) | (%) | (%) | p-value |
| **Census Region** | | | | | |
| Midwest | 13.3 | 16.9 | 5.3 | 21.2 | <0.001 |
| Northeast | 27.0 | 16.9 | 27.5 | 21.2 | |
| South | 34.8 | 44.6 | 64.1 | 42.3 | |
| West | 25.0 | 21.5 | 3.1 | 15.4 | |
| **Location** | | | | | |
| Rural | 3.9 | 7.7 | 4.6 | 5.8 | NS |
| Urban | 96.1 | 92.3 | 95.4 | 94.2 | |
| **Teaching** | | | | | |
| No | 38.7 | 50.8 | 32.7 | 55.8 | <0.05 |
| Yes | 61.3 | 49.2 | 67.3 | 44.2 | |
| **Bed Count** | | | | | |
| <300 | 15.6 | 15.4 | 2.1 | 13.5 | <0.001 |
| 301 - 500 | 38.2 | 49.2 | 26.9 | 50.0 | |
| >501 | 46.1 | 35.4 | 71 | 36.5 | |

AVR: aortic valve replacement; MVR: mitral valve replacement; MVRepair: mitral valve repair; RT: right thoracotomy

**Table 3.** Hospital Characteristics After Matching by Valvular Procedure - continued

| | MVR | | | | |
|---|---|---|---|---|---|
| | Sternal | | RT | | |
| | Visits (n= 217) | Hospitals (n = 50) | Visits (n= 217) | Hospitals (n = 41) | |
| **Category** | **(%)** | **(%)** | **(%)** | **(%)** | **p-value** |
| **Census Region** | | | | | |
| Midwest | 10.7 | 16 | 5.5 | 19.5 | <0.001 |
| Northeast | 11.7 | 14 | 15.1 | 24.4 | |
| South | 39.8 | 46 | 75.2 | 36.6 | |
| West | 37.9 | 24 | 4.1 | 19.5 | |
| **Location** | | | | | |
| Rural | 1.8 | 8.0 | 2.4 | 4.9 | NS |
| Urban | 98.2 | 92.0 | 97.6 | 95.1 | |
| **Teaching** | | | | | |
| No | 42.7 | 50.0 | 18.4 | 48.8 | <0.05 |
| Yes | 57.3 | 50.0 | 81.7 | 51.2 | |
| **Bed Count** | | | | | |
| <300 | 7.8 | 14.0 | 3.7 | 17 | <0.001 |
| 301 - 500 | 43.2 | 50.0 | 13.3 | 43.9 | |
| >501 | 49.0 | 36.0 | 83.0 | 39.0 | |
| | **MVRepair** | | | | |
| | **Sternal** | | **RT** | | |
| | **Visits (n= 309)** | **Hospitals (n =43)** | **Visits (n= 309)** | **Hospitals (n = 35)** | |
| **Category** | **(%)** | **(%)** | **(%)** | **(%)** | **p-value** |
| **Census Region** | | | | | |
| Midwest | 7.7 | 14 | 4.4 | 17.1 | <0.001 |
| Northeast | 16.4 | 16.3 | 21.0 | 31.4 | |
| South | 38.0 | 41.9 | 69.2 | 28.6 | |
| West | 38.0 | 27.9 | 5.3 | 22.9 | |
| **Location** | | | | | |
| Rural | 3.7 | 9.3 | 1.2 | 5.7 | <0.05 |
| Urban | 96.4 | 90.7 | 98.8 | 94.3 | |
| **Teaching** | | | | | |
| No | 45.6 | 48.8 | 12.4 | 45.7 | <0.001 |
| Yes | 54.4 | 51.2 | 87.6 | 54.3 | |
| **Bed Count** | | | | | |
| <300 | 11.0 | 18.6 | 1.5 | 8.6 | <0.001 |
| 301 - 500 | 46.0 | 44.2 | 8.3 | 45.7 | |
| >501 | 43.1 | 37.2 | 90.2 | 45.7 | |

AVR: aortic valve replacement; MVR: mitral valve replacement; MVRepair: mitral valve repair; RT: right thoracotomy

**Table 4.** Least Square (LS) Means for OR Cost per Minute by Surgical Approach within Valvular Procedure

| | AVR | | | MVR | | | MVRepair | | |
|---|---|---|---|---|---|---|---|---|---|
| | Sternal | RT | p-value | Sternal | RT | p-value | Sternal | RT | p-value |
| **OR cost per Minute** | | | | | | | | | |
| **Estimate** | $26.49 | $25.16 | <0.01 | $25.66 | $25.00 | NS | $25.17 | $24.40 | NS |

P-value shown compares Sternal vs. RT for the specific valvular procedure.

LS means adjusted for region, teaching status, urban status, and number of beds. OR cost per minute was modeled using generalized linear models with a gamma distribution and log link due to the inherent distribution of cost data.

LS: least square; AVR: aortic valve replacement; MVR: mitral valve replacement; MVRepair: mitral valve repair; RT: right thoracotomy; OR: operating room

### DISCUSSION

Health service researchers interested in economic evaluations pertaining to heart valve procedures have lacked a reliable source for estimating the value of OR time to the hospital. This study has attempted to fill this gap, with the goal of promoting more refined and accurate estimates of heart valve surgery OR cost. Additionally, this approach may provide a more accurate method for assessing the value of potential savings associated with new technologies or techniques that may reduce OR time and costs relative to the current standard of care.

While the OR costs per minute differed across procedures by surgical approach, they were modest in magnitude. Moreover, the mean OR cost per minute for the lowest-cost procedure (RT AVR) was $25.16, just $1.33 less than the highest mean OR cost per minute (AVR Sternal). The OR costs per minute for MVR and MVRepair were between the AVR Sternal and AVR RT procedures and the differences between surgical approaches were not significant. Given the tight grouping of OR cost per minute observed between these procedures, the overall mean OR cost per minute of $25.99 may be a reasonable and sufficient estimate for heart valve surgeries.

Valve replacement surgery is lengthy, regardless of procedure type or surgical approach. Multiplying OR cost per minute by average OR time for each procedure, we find that total OR costs were $8,076, $9,542, and $9,282 for AVR, MVR and MVRepair, respectively. MVR and MVRepair are more complex and therefore time intensive procedures compared to AVR. These results indicate that the total OR cost for valve replacement surgery is substantial for each of these procedures. As a result, inefficiencies during a procedure may lead to further significant cost increases. In the current cost-conscious health care environment, it is important to pay attention to time saving measures, especially with lengthy procedures such as heart valve surgeries.

This study had some noteworthy limitations. First, the data used to identify OR time came from a hospital system administrative database, rather than a database expressly used to track OR time and resources. However, the database is used to track resource utilization for billing purposes and it is incumbent on the hospitals to track resource utilization accurately. While, the hospital OR costs are estimated precisely, different institutions may have placed various items into different cost centers and this may be a source of discrepancy. However, these limitations are inherent to the data source and could be rationalized to impact all cohorts similarly. Although the Premier database contains nearly 600 hospitals and has a similar characteristic profile reported by the American Hospital Association Annual Survey,[16] the results may not be applicable to all settings. Additionally, we could not control for the effect of surgeon characteristics within these analyses. Surgeon characteristics such as experience and procedure volume may influence the level of OR resources consumed and impact the OR cost per minute.

### CONCLUSIONS

This study proposed and implemented a novel method to estimate the OR cost per minute from a multi-institution database for valvular surgeries. The findings indicate that the hospital cost per minute in the OR may range between $25.00 and $26.50 with an overall mean of $25.99. Given the paucity of data and wide range of previously reported OR costs per minute, this study provides a useful method and parameter for use in future economic evaluations when assessing the trade-offs between time-saving technologies versus their upfront costs for heart valve surgeries.

### CONFLICT OF INTEREST DECLARATION

Financial support for this study was provided by Edwards Lifesciences, Inc. EG is a consultant to Edwards Lifesciences, Inc. and Medtronic, plc. and holds Intellectual property with Edwards Lifesciences and Medtronic, plc. MM is an employee of the study sponsor, Edwards Lifesciences, Inc. PM is an employee of and JR is a consultant of CTI Clinical Trial and Consulting Services which is a paid consultant to Edwards Lifesciences, Inc.
